# Bright, but allergic and neurotic? A critical investigation of the “overexcitable genius” hypothesis

**DOI:** 10.3389/fpsyg.2022.1051910

**Published:** 2022-12-23

**Authors:** Jonathan Fries, Tanja Gabriele Baudson, Kristof Kovacs, Jakob Pietschnig

**Affiliations:** ^1^Department of Developmental and Educational Psychology, Faculty of Psychology, University of Vienna, Vienna, Austria; ^2^Department of Human Sciences, Institute of Psychology, Vinzenz Pallotti University, Vallendar, Germany; ^3^Institute for Globally Distributed Open Research and Education (IGDORE), Vallendar, Germany; ^4^Department of Science and Research, Mensa in Germany, Cham, Germany; ^5^Institute of Psychology, ELTE Eotvos Lorand University Budapest, Budapest, Hungary

**Keywords:** intelligence, cognitive ability, health, mental health, giftedness, overexcitability, theory of positive disintegration

## Abstract

**Introduction:**

Higher intelligence has been associated with improved health and longevity. However, recent findings have claimed that exceptional intelligence may come at a cost. Individuals at the upmost end of the intelligence distribution are reported to be disproportionately afflicted by a set of stress-related physical and mental health conditions: so-called *overexcitabilities*. Few accounts have investigated this issue and no studies are available for non-US samples yet. Here, we aimed to replicate and extend previous work by examining hitherto unaddressed overexcitabilities in a European high-IQ sample.

**Methods:**

We carried out a preregistered survey among members of MENSA, the world’s largest high-IQ society. In total, 615 (307 male) members from Austria, Germany, Hungary, Switzerland, and the United Kingdom participated.

**Results and Discussion:**

Compared to the general population, our sample exhibited considerably elevated prevalences in autism spectrum disorders (risk ratio/*RR* = 2.25), chronic fatigue syndrome (*RR* = 5.69), depression (*RR* = 4.38), generalized anxiety (*RR* = 3.82), and irritable bowel syndrome (*RR* = 3.76). Contrary to previous accounts, neither asthma, allergies, nor autoimmune diseases were elevated. We show that this subsample of intellectually gifted persons faces specific health challenges compared to the general population. The reasons for this remain speculative, as we find little evidence for previously proposed immunological explanations. However, it is possible that the effects are caused by sample selectiveness (i.e., membership in a high-IQ society) rather than high IQ itself.

## 1. Introduction

Intelligence, often used interchangeably with “cognitive” or “intellectual ability,” is a broad capacity that enables us to understand our surroundings, solve problems, predict, plan, and reason (e.g., Hunt, 2011). High intelligence is usually considered a beneficial trait because it facilitates educational (Becker et al., 2019) and professional success (Kuncel et al., 2014), and correlates with attractiveness to potential romantic partners (Prokosch et al., 2009) as well as career earnings (Furnham and Cheng, 2013). Persons of high intelligence are often described using the term “intellectually gifted”. While virtually all definitions of intellectual giftedness agree upon exceptional cognitive ability as a central component, there is no consensus which, if any, additional factors should be considered (Carman, 2013). Some concepts regard creativity (e.g., Renzulli, 2005) or achievement motivation (e.g., Tannenbaum, 2003) as integral parts of giftedness, while others emphasize well-being and the fulfilment of one’s potential (e.g., Drigas et al., 2017).

Cognitive ability has been found to be associated with health and longevity, with more intelligent individuals exhibiting more favorable physical and mental health as well as longer lifespans, even when socioeconomic and educational variables are held constant (Deary et al., 2021; Fries and Pietschnig, 2022). Contrasting accounts suggest that high intelligence comes at a significant cost: intellectually gifted individuals are claimed to exhibit higher rates of interpersonal maladjustment (Matta et al., 2019) and poorer emotional competence (Guénolé et al., 2013) compared to non-gifted individuals (but see also Martin et al., 2010; Zeidner and Shani-Zinovich, 2011).

This line of research is commonly summarized under the term *disharmony hypothesis* of giftedness, highlighting the less desirable associations of exceptional intellectual capability. The emphasis of the alleged negative aspects of giftedness also informs the so-called “mad genius” stereotype, which is common among education professionals (Baudson and Preckel, 2016) as well as the general population (Baudson, 2016).

Recently, evidence has emerged suggesting that the relationship between intelligence and health – which has typically been described as linear across the entire intelligence spectrum (e.g., Wraw et al., 2015; Brown et al., 2021) – might in fact be curvilinear. In a community survey among members of the US-based chapter of MENSA, probably the world’s largest international association of intellectually gifted persons with more than 145,000 members (MENSA International, 2022), Karpinski et al. (2018) found substantially elevated levels of asthma, allergies, attention deficit disorders (ADD/ADHD), autism spectrum disorders (ASD), autoimmune diseases, depression, and anxiety disorders compared to the general population. The authors proposed that a psychoneuroimmunological mechanism may be responsible for the observed higher prevalences. Following their rationale, highly intelligent persons are physically and mentally “overexcitable” – i.e., they tend towards higher awareness for and reactivity to their surroundings. This supposedly puts the sympathetic nervous system in a state of permanent activation, which, in turn, can cause the immune system to respond in dysfunctional ways. Suggested physiological outcomes of this theoretical pathway are autoimmune diseases and allergies (Karpinski et al., 2018). Autism spectrum disorders are also suspected to be a possible consequence of chronic neuroinflammatory responses by the immune system (Theoharides et al., 2013). From a mental health perspective, the over-reactivity to internal and external stimuli may predispose affected individuals to ruminate and worry, thus leading to mood (e.g., depression or bipolar disorder), anxiety, and attention deficit disorders (ADD/ADHD; Karpinski et al., 2018).

Because “overexcitability” is a term with varying definitions, it is critical to briefly elaborate on the concept. It was coined by the Polish psychologist Kazmierz Dąbrowski in his Theory of Positive Disintegration. Based on his observations in gifted persons, he posited that intellectual giftedness comes with increased physical and psychological excitability which enhances the experience intensity of inner tensions and conflicts (Dąbrowski, 1966). Based on his theory, a five-factor model of overexcitability was later conceptualized. It is composed of psychomotor, sensual, imaginational, intellectual, and emotional overexcitabilities (Falk et al., 1999). Psychomotor overexcitability represents a person’s inclination for physical movement. Sensual overexcitability represents a person’s disposition to perceive the world through their senses, like touch or vision. Intellectual overexcitability represents a person’s affinity for gaining new knowledge through various ways. Imaginational overexcitability represents a person’s disposition to immerse themselves in fantasies or stories. Emotional overexcitability represents a person’s level of emotional experience and expression (Bouchet and Falk, 2001). Karpinski et al. (2018) depart from Dąbrowski’s concept as the latter does not indicate physical or mental illness. Therefore, the overexcitability concept of Karpinski et al. (2018) can be interpreted as an attempt to extend Dąbrowski’s notion of overexcitability to the area of physical and mental health. The original overexcitability construct has been criticized for lacking empirical support and not containing incremental information beyond personality constructs such as the Five Factor Model (Limont et al., 2014; Vuyk et al., 2016a,b).

Here, we intended to replicate and extend the findings of Karpinski et al. (2018) in a European sample with the broader goals to investigate the patterns of physical and mental health in intellectually gifted individuals and to determine whether *overexcitability* is a useful construct in scientific practice. We hypothesized elevated rates of ADD/ADHD, autism spectrum disorder, asthma, allergies, autoimmune disorders, depression, generalized anxiety disorders and additional conditions that have exhibited etiological associations to psychoneuroimmunology (e.g., Haroon et al., 2012; Irwin, 2015; Moraes et al., 2018).

We suspected that the way in which individuals cope with emotionally challenging situations may be an important factor in the proposed psychoneuroimmunological mechanism. Previous research has shown that a head-on approach to dealing with emotional stress is more conducive to mental health as opposed to an avoidant approach (Seiffge-Krenke and Klessinger, 2000). Consequently, we hypothesized that persons that exhibit more problem-focused coping styles exhibit less physical and mental health conditions than people exhibiting an avoidant coping style. In other words, coping style could moderate the relationship between intellectual giftedness and susceptibility to overexcitabilities.

Confirmatory analyses for this study were preregistered before data collection.^1^ In additional exploratory analyses, we intended to investigate how personality interacts with the relationship between health and intellectual giftedness to determine if the concept of overexcitability can provide additional insights that are not captured by the more established concept of personality. We also aimed to examine the conceptual links between Dąbrowski’s overexcitability construct and the more pathology-centered interpretation proposed by Karpinski et al. (2018).

## 2. Materials and methods

### 2.1. Design and sample

The current study was an observational single-group survey. It was conceptualized as a preregistered replication and extension of Karpinski et al. (2018) and was preregistered prior to data collection.^1^

Following Karpinski et al.’s (2018) approach, we surveyed members of MENSA, an international society exclusively for individuals scoring in the top 2% of the general population on a standardized test of intelligence (MENSA International, 2020). We invited all members aged 18 years and above from the chapters in Austria, Germany, Hungary, Switzerland, and the United Kingdom via mailing lists and Facebook groups.

In sum, 617 participants (308 women) completed the survey. Descriptive information on participant demographics is summarized in Tables 1 and 2.

**Table 1:** Descriptive statistics for the current sample.

	*Mean*	*Mdn*	*SD*	*IQR*
Participant age				
Overall	47.98	48.00	14.91	21.00
Women	46.17	45.00	14.32	18.00
Men	49.98	50.00	15.15	24.00
Intelligence test				
Time passed since IQ test	14.13	9.00	13.33	20.00
^1^Annual net income in Euro				
Austria	32,642.96	30,000.00	20,100.48	24,500.00
Switzerland	59,697.00	61,300.00	35,800.16	38,664.00
Germany	45,365.71	47,460.00	21,456.89	22,500.00
Hungary	28,388.97	16,200.00	67,549.76	12,474.00
United Kingdom	52,511.75	40,950.00	56,345.55	36,055.00
BMI				
BMI	26.68	25.30	7.74	6.92

1 For Switzerland, Hungary, and the UK, annual income was converted from the respective regional currencies to Euro using the exchange rate of December 17, 2021.

**Table 2:** Frequencies for sociodemographic variables.

	Frequency	Percentage
Country of residence		
AT	39	6.32
CH	40	6.48
DE	119	19.29
HU	76	12.32
UK	343	55.59
Occupation		
Armed forces occupation	3	0.49
Clerical support worker	52	8.43
Craft and related trades worker	11	1.78
Elementary occupation	4	0.65
Manager	96	15.56
Plant and machine operator	3	0.49
Professional	302	48.95
Service and sales worker	21	3.40
Skilled agricultural, forestry or fishery worker	5	0.81
Technician or associate professional	81	13.13
Not answered	39	6.32
Education		
No degree	17	2.76
Post-secondary education	104	16.86
Secondary education	40	6.48
Bachelor’s degree or equivalent	153	24.80
Master’s degree or equivalent	229	37.12
Doctoral degree/PhD	69	11.18
Not answered	5	0.81
Intelligence test results		
> 130	165	26.74
> 135	238	38.57
I do not recall	214	34.68

### 2.2. Measures

Data for the current study were collected through an online survey comprising assessments of socio-demographic data, a physical and mental health section as well as personality and coping questionnaires. The questionnaire took about 45 to 60 min to complete.

In the physical and mental health section, participants were given a list of physical and mental health conditions. For each of these conditions, we asked participants whether they were currently diagnosed with the respective condition or whether they suspected to be suffering from it. Subsequently, diagnosed and the combination of diagnosed and suspected conditions were analyzed as separate categories. This questionnaire extended the original questionnaire used by Karpinski et al. (2018), which we obtained *via* personal communication with the authors (R. Karpinski, personal communication, September 4, 2020). It was extended by 26 additional physical and mental health conditions to the 13 conditions contained in the published report by Karpinski et al. (2018), resulting in a total of 39 conditions. For a full list and detailed comparison with the original study, see Table 3. For the added conditions, psychoneuroendocrinological or stress-related mechanisms have been proposed (e.g., Haroon et al., 2012; Irwin, 2015; Moraes et al., 2018) and consequently, following the rationale suggested by Karpinski et al. (2018), were expected to exhibit elevated rates in the current sample.

**Table 3:** Prevalences, frequencies, risk ratios and *p*-values for all conditions that were compared against the general population.

	Population rate	Observed rate (diagnosed)	Cases (diagnosed)	*p* (diagnosed)	*RR* (diagnosed)	Observed rate (combined)	Cases (combined)	*p* (combined)	*RR* (combined)
ADHD or ADD	4.70	3.41	21	0.95	0.73	9.43	58	0.01	2.01
Alcoholism	0.70	0.65	4	0.62	0.93	3.58	22	0.01	5.11
Alzheimer’s disease	5.05	0.00	0	1.00	0.00	0.16	1	1.00	0.03
Amyotrophic lateral sclerosis (ALS)	0.01	0.16	1	0.05	18.07	0.33	2	0.01	36.13
Asperger’s syndrome	0.03	3.25	20	0.01	98.55	13.17	81	0.01	399.11
Asthma	6.20	7.80	48	0.06	1.26	12.20	75	0.01	1.97
Autism	0.94	2.11	13	0.01	2.25	6.67	41	0.01	7.09
Autoimmune disease(s)	5.29	6.50	40	0.11	1.23	8.94	55	0.01	1.69
Back pain	46.10	11.06	68	1.00	0.24	28.13	173	1.00	0.61
Bipolar disorder	1.00	0.98	6	0.58	0.98	2.44	15	0.01	2.44
Borderline personality disorder	2.00	0.81	5	0.99	0.41	2.28	14	0.35	1.14
Cancer	1.01	3.09	19	0.01	3.07	4.07	25	0.01	4.04
Chronic fatigue syndrome	0.20	1.14	7	0.01	5.69	6.83	42	0.01	34.15
Dementia	7.10	0.00	0	1.00	0.00	0.49	3	1.00	0.07
Depression	3.90	17.07	105	0.01	4.38	27.32	168	0.01	7.00
Dyscalculia	5.00	0.00	0	1.00	0.00	0.98	6	1.00	0.20
Dyslexia	3.80	1.63	10	1.00	0.43	5.04	31	0.07	1.33
Dyspraxia	5.50	0.33	2	1.00	0.06	0.98	6	1.00	0.18
Environmental allergies	14.80	13.50	83	0.83	0.91	29.76	183	0.01	2.01
Epilepsy	0.91	0.49	3	0.92	0.54	0.81	5	0.66	0.89
Fibromyalgia	4.70	0.33	2	1.00	0.07	1.63	10	1.00	0.35
Food allergies	4.70	6.18	38	0.06	1.31	13.17	81	0.01	2.80
Food sensitivities	25.50	4.55	28	1.00	0.18	21.14	130	0.99	0.83
Generalized anxiety	2.00	7.64	47	0.01	3.82	19.51	120	0.01	9.76
Illegal drug abuse	7.10	0.49	3	1.00	0.07	1.95	12	1.00	0.27
Irritable bowel syndrome	1.34	5.04	31	0.01	3.76	13.17	81	0.01	9.83
Lactose intolerance	14.00	4.23	26	1.00	0.30	14.31	88	0.43	1.02
Migraine	10.40	6.50	40	1.00	0.63	14.96	92	0.01	1.44
Narcissistic personality disorder	7.05	0.98	6	1.00	0.14	2.44	15	1.00	0.35
Obesity	24.00	7.97	49	1.00	0.33	19.51	120	1.00	0.81
Obsessive compulsive disorder	0.70	1.14	7	0.14	1.63	6.18	38	0.01	8.83
Parkinson’s disease	1.40	0.16	1	1.00	0.12	0.33	2	1.00	0.23
Pervasive developmental disorder	0.60	0.00	0	1.00	0.00	0.16	1	0.98	0.27
Phobia(s)	3.50	1.30	8	1.00	0.37	7.15	44	0.01	2.04
Psychopathy	0.60	0.00	0	1.00	0.00	0.81	5	0.31	1.36
Schizophrenia	0.33	0.33	2	0.60	0.99	0.65	4	0.15	1.97
Sleep disorder	9.40	4.07	25	1.00	0.43	18.21	112	0.01	1.94
Social anxiety	4.40	5.04	31	0.24	1.15	19.35	119	0.01	4.40
Vertigo	15.80	1.79	11	1.00	0.11	6.67	41	1.00	0.42

Note: *p*-values correspond to binomial tests of the sample prevalences against the population prevalences; *RR*s are calculated using the population prevalence as reference.

In addition, participants were asked to recall the percentile they achieved in their MENSA admission test relative to the general population and to assign themselves to either the top 2, corresponding to an IQ test score over 130, or top 1 percent category, corresponding to an IQ test score over 135. If participants were unable to recall the test result, they were asked to respond, “I do not recall.” Table 2 contains frequencies on the MENSA IQ test results.

The Brief-COPE inventory (Carver, 1997), a 28-item questionnaire inquiring about ways in which participants cope with various situations, was applied to assess the coping strategies employed by participants. Respondents were asked to estimate how often they engaged in a specific behavior in the past using a four-point Likert scale. Following the author’s instructions, sum scores for problem-focused coping (eight items), emotion-focused coping (12 items), and avoidant coping (eight items) were computed (Carver, 1997). Problem-focused coping can be considered an adaptive coping strategy, while emotion-focused and avoidant coping can be considered maladaptive (Carver, 1997). Factors exhibited modest to adequate internal consistency, with Cronbach’s α = 0.61 (Avoidant coping), α = 0.71 (Emotion-focused coping), and α = 0.80 (Problem-focused coping). Furthermore, scores for individual coping strategies were computed by averaging the two items pertaining to each subscale.

The Overexcitability Questionnaire II (OEQ-II; Falk et al., 1999) is a 50-item inventory assessing psychomotor, sensual, imaginational, intellectual, and emotional overexcitabilities. Respondents rate their agreement with a statement on a five-point Likert scale ranging from “not at all like me” to “very much like me.” From individual items, five factor scores are computed, Psychomotor, Sensual, Imaginational, Intellectual, and Emotional overexcitability. The factors exhibited adequate internal consistency, with Cronbach’s α ranging from 0.84 to 0.89.

The HEXACO-60 (Ashton and Lee, 2009) measures the personality factors honesty-humility, emotionality, extraversion, agreeableness, conscientiousness, and openness to experience *via* 60 items (10 per subscale). Respondents rate their agreement with a statement on a five-point Likert scale. Each factor is subdivided into 4 facets. The factors exhibited modest to adequate internal consistency, with Cronbach’s α ranging from 0.67 to 0.82.

The questionnaire for the current study was available in the respective languages of the participating MENSA chapters, English, German, and Hungarian. While pre-existing translations were available for the HEXACO personality questionnaire and the Brief-COPE, this was neither the case for the OEQ-II nor for the part we adapted from Karpinski et al. (2018). All sections for which no translations were available in the literature were translated from English into German and Hungarian by the authors.

### 2.3. Data analysis

Following Karpinski et al.’s (2018) approach, we compared physical and mental health conditions prevalences in the current sample with prevalences in the general population. These reference values were taken from the scientific literature for each condition. Studies were selected based on largest available sample size, given that the geographical region where the respective studies were conducted matched at least one of the participating chapters’ countries. Table 3 shows a full list of all conditions and sources of reference data.

For each condition, we performed two comparisons. First, we examined whether rates of clinically diagnosed conditions were elevated compared to the general population. Second, we examined whether rates of diagnosed and suspected conditions combined (henceforth: “combined”) were elevated.

Statistical comparisons of the sample versus population prevalences were carried out using one-tailed binomial tests. For each comparison we calculated risk ratios to allow assessments of effect size.

For comparisons of the number of conditions between the two IQ levels (i.e., top 1 percent vs. top 2 percent) assessed in the questionnaire, we used two-tailed, two-sample Wilcoxon tests.

Correlations and partial correlations were computed using the Spearman method because not all variables were measured on an interval scale.

The over-reliance on *p*-values in the interpretation of statistical analyses is well-known to frequently produce false-positive results (Simmons et al., 2011). However, adjustments that are intended to counteract type-I error inflation are plagued by serious methodological concerns (e.g., Perneger, 1998; Nakagawa, 2004). Thus, in the present study, we chose to use *p*-values only as a non-triviality threshold. To determine whether effects were meaningful, we used effect sizes. As a threshold for non-triviality, we adopted a *p*-value of 0.01. As benchmarks for effect size, we followed the recommendations by Cohen (1988). As benchmarks for Cronbach’s α, we referred to the remarks by Nunnally and Bernstein (1994).

For all analyses, R 4.1.0 was utilized (R Core Team, 2022). Figures were created using the R package ggplot2 (Wickham, 2016).

The datasets generated and/or analyzed during the current study are not publicly available due to data protection considerations, but are available in truncated form from the corresponding author on reasonable request.

This study was carried out in accordance with the principles laid out in the Declaration of Helsinki (World Medical Association, 2013). The research design was strictly observational, no interventions were performed, and no risks were involved for participants. Informed consent was gathered from all participants involved in this study. In accordance with national law and with the ethical guidelines of the authors’ research institutions, ethical approval was not required for this study (§ 30 Universitätsgesetz, 2002; University of Vienna, 2017).

## 3. Results

**Figure 1:**
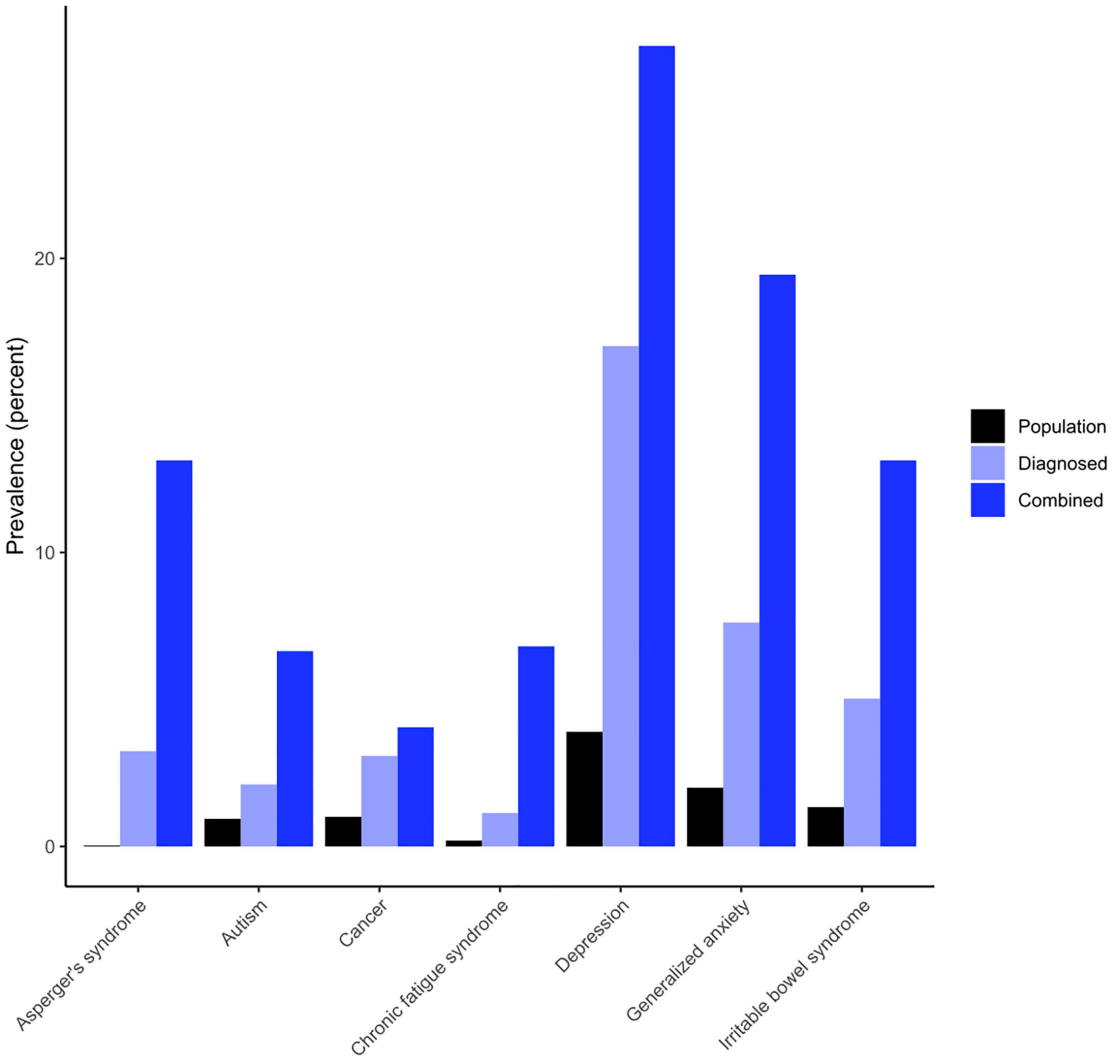
Bar chart for all conditions that exhibited elevated sample prevalences.

In our sample, we found elevated rates of various physical and mental health conditions; see Figure 1 for a bar chart of selected sample and population prevalences, and Table 4 for detailed results for sample-population comparisons. For each condition, participants were asked whether a clinician had diagnosed them or whether they suspected to suffer from it, in the absence of a clinical diagnosis. In diagnosed conditions, we found significantly elevated rates compared to general population prevalences among 7 out of 39 categories: amyotrophic lateral sclerosis (ALS), Asperger’s syndrome, autism, cancer, chronic fatigue syndrome, depression, generalized anxiety, and irritable bowel syndrome. In combined conditions, prevalences were considerably higher for most conditions and we found significantly elevated rates in 20 out of 39 conditions.

**Table 4:** Prevalences for all examined conditions with literature references.

Condition	^1^Prevalence US (percent)	Prevalence Europe (percent)	Reference Europe
ADHD/ADD	4.10	4.70	Philipsen and Döpfner (2020)
^3^Alcoholism	-	0.70	Alonso et al. (2000)
^3^ALS	-	0.01	Hardiman et al. (2017)
^3^Alzheimer’s disease	-	5.05	Niu et al. (2017)
Asperger’s syndrome	0.03	0.03	Isaksen et al. (2013)
Asthma	7.40	6.20	Steppuhn et al. (2017)
Autism	1.00	0.94	Baron-Cohen et al. (2009)
Autoimmune disease(s)	8.00	5.29	Eaton et al. (2007)
Back pain	-	46.10	Farioli et al. (2014)
Bipolar disorder	2.60	1.00	Pini et al. (2005)
^3^Borderline personality disorder	-	2.00	Bode et al. (2017)
^3^Cancer	-	1.01	Zentrum für Krebsregisterdaten and Gesellschaft der epidemiologischen Krebsregister in Deutschland e.V. (2021)
^2^^,^^3^Chronic dental issues	-	-	-
^2^^,^^3^Chronic ear infections during childhood	-	-	-
^3^Chronic fatigue syndrome	-	0.20	Nacul et al. (2011)
^3^Dementia	-	7.10	Bacigalupo et al. (2018)
Depression	6.70	3.90	Alonso et al. (2000)
^2^^,^^3^Digestive issues	-	-	-
Dyscalculia	-	5.00	Fischbach et al. (2013)
^3^Dyslexia	-	3.80	Fischbach et al. (2013)
Dyspraxia	-	5.50	Blank et al. (2019)
Environmental allergies	10.60	14.80	Langen et al. (2013)
^3^Epilepsy	-	0.91	Hamer et al. (2012)
^3^Fibromyalgia	-	4.70	Branco et al. (2010)
Food allergies	3.70	4.70	Bergmann et al. (2016)
^3^Food sensitivities	-	25.50	Bergmann et al. (2016)
General anxiety	3.10	2.00	Lieb et al. (2005)
^3^Illegal drug abuse	-	7.10	Schneider et al. (2020)
^3^Irritable bowel syndrome	-	1.34	Häuser et al. (2019)
^2^Joint pain	-	-	-
Lactose intolerance	20.00	14.00	Storhaug et al. (2017)
Migraine	-	10.40	Porst et al. (2020)
^3^Narcissistic personality disorder	-	7.05	Gawda and Czubak (2017)
Obesity	-	24.00	Duncan and Toledo (2018)
Obsessive compulsive disorder	1.00	0.70	Adam et al. (2012)
^3^Parkinson’s disease	-	1.40	Pringsheim et al. (2014)
^3^Pervasive developmental disorder	-	0.73	Elsabbagh et al. (2012)
Phobia(s)	-	3.50	Alonso et al. (2000)
^3^Psychopathy	-	0.60	Coid et al. (2009)
^3^Schizophrenia	-	0.33	Simeone et al. (2015)
Sleep disorder	-	9.40	Marschall et al. (2017)
Social anxiety	6.80	4.40	Ohayon and Schatzberg (2010)
^4^Sociopathy	-	-	-
^3^Vertigo	-	15.80	Wiltink et al. (2009)
^2^^,^^3^Working memory issues	-	-	-

Estimates of population prevalences to which the current sample was compared.

1 Prevalences as taken from Karpinski et al. (2018).

2 Contained in original questionnaire by Karpinski et al. (2018; R. Karpinski, personal communication, September 4, 2020), but not reported in the published study. We were unable to find reliable comparison data for this category because the condition is not adequately defined.

3 Condition was contained in the original questionnaire as provided to us by the corresponding author of Karpinski et al. (2018; R. Karpinski, personal communication, September 4, 2020), but not reported in the published study. Therefore, no prevalence was available.

4 The term “Sociopathy” is not sufficiently distinct from the term “Psychopathy.” Therefore, only the latter category was used here.

Participants that scored within the top 1 percent of the intelligence distribution did not differ from participants that scored within the top 2 percent regarding the number of diagnosed (*W* = 20,818, *p* = 0.27, *d* = 0.11) or combined conditions (*W* = 19,146, *p* = 0.67, *d* = 0.06).

**Figure 2:**
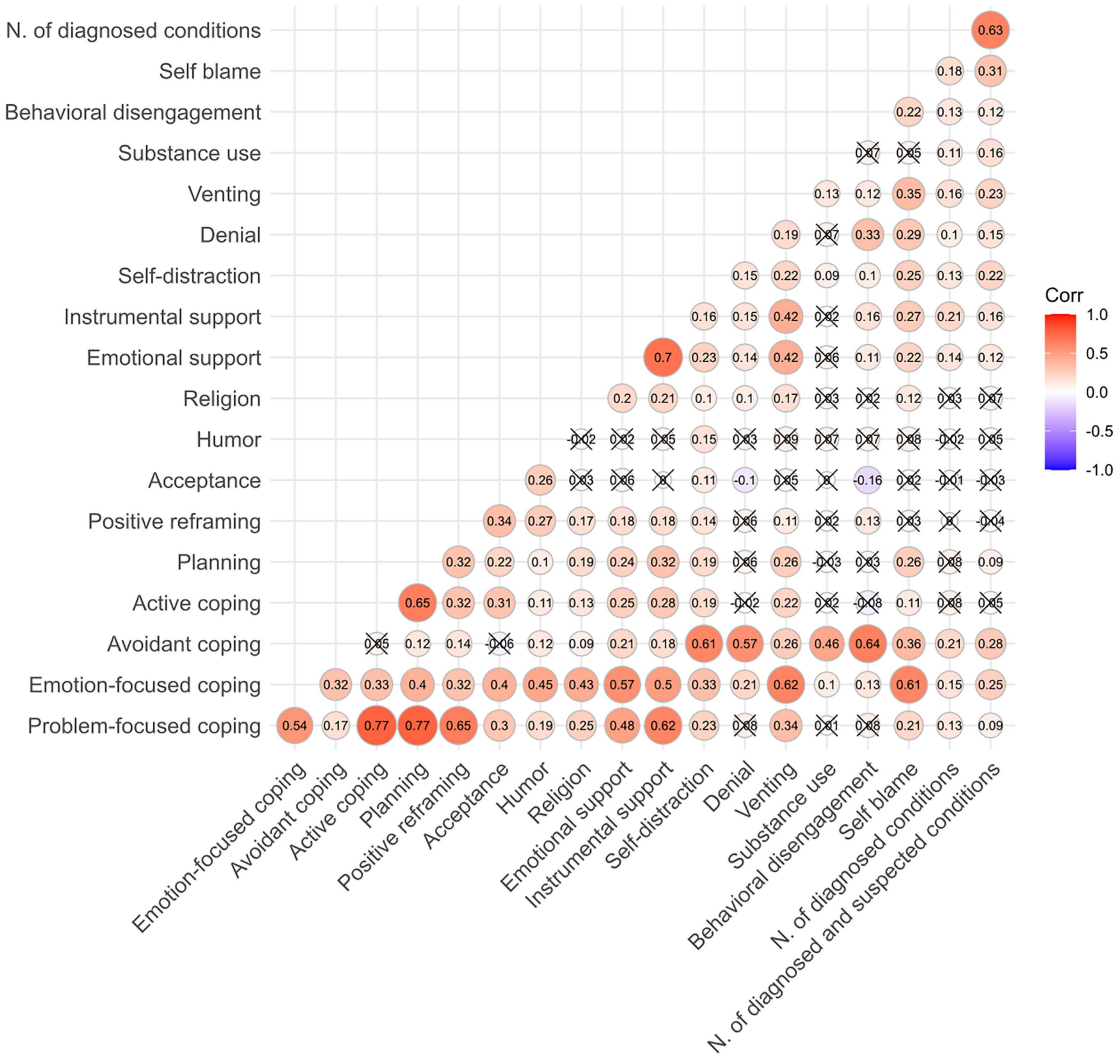
Correlation heatmap for bivariate Spearman correlations of the number of physiological and psychological overexcitabilities and coping strategies assessed using the Brief COPE.

The number of diagnosed conditions correlated modestly but significantly with a range of coping strategies; Figure 2 shows a correlation heatmap for bivariate Spearman correlations of the number of reported conditions and coping strategies assessed using the Brief-COPE. Among the factor scores, avoidant coping exhibited the highest correlations with the number of reported conditions (*r* = 0.21, *p* < 0.01), followed by emotion-focused (*r* = 0.15, *p* < 0.01), and problem-focused coping (*r* = 0.13, *p* < 0.01). This pattern emerged analogously, but more pronouncedly, for correlations with combined conditions (avoidant coping *r* = 0.28, *p* < 0.01; emotion-focused coping *r* = 0.25, *p* < 0.01; problem-focused coping *r* = 0.09, *p* = 0.03).

**Figure 3:**
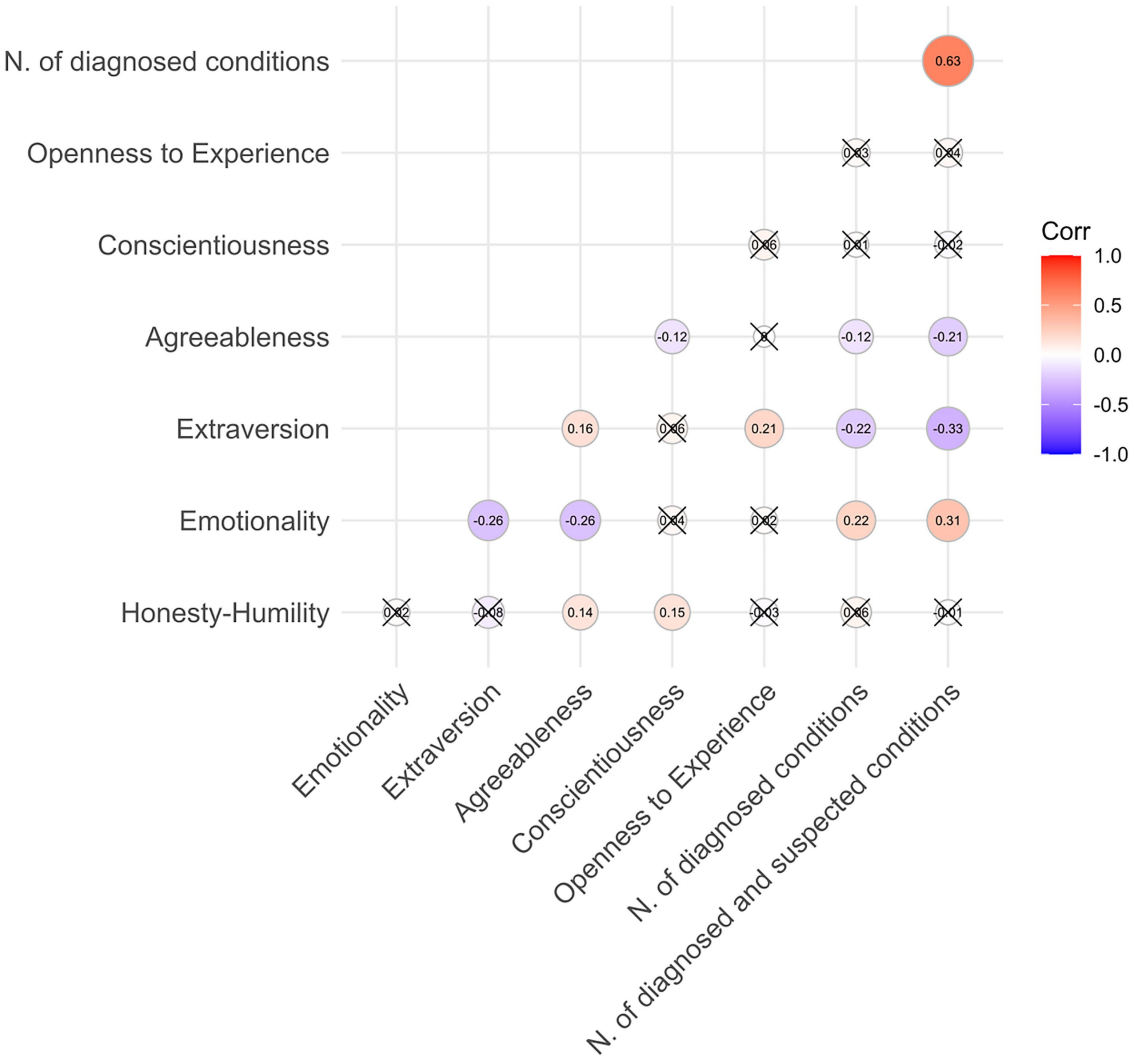
Correlation heatmap for bivariate Spearman correlations of the number of physiological and psychological health conditions and the six HEXACO personality dimensions.

In explorative analyses, we investigated the relations of physical and mental health with personality; see Figure 3 for a correlation heatmap of associations between HEXACO personality factors and self-reported physical and mental health conditions. The number of diagnosed conditions correlated moderately but significantly with the HEXACO factors Extraversion (*r* = −0.22, *p* < 0.01) and Agreeableness (*r* = −0.12, *p* < 0.01), indicating that lower values in these personality factors were associated with a higher number of diagnosed conditions. Conversely, higher Emotionality was associated with a higher number of diagnosed conditions (*r* = 0.22, *p* < 0.01). For combined conditions, even stronger effects in identical directions were observed.

To investigate the proposed link between rumination and worry (Karpinski et al., 2018), we examined correlations with the HEXACO factor Emotionality on a facet level. For the number of diagnosed conditions, the strongest effect sizes were found in Anxiety (*r* = 0.23, *p* < 0.01), followed by Dependence (*r* = 0.13, *p* < 0.01), Fearfulness (*r* = 0.13., *p* < 0.01) and Sentimentality (*r* = 0.13, *p* < 0.01). This correlation was notably larger for the number of combined conditions (*r* = 0.37, *p* < 0.01), indicating a higher number of combined conditions for more anxious individuals.

**Figure 4:**
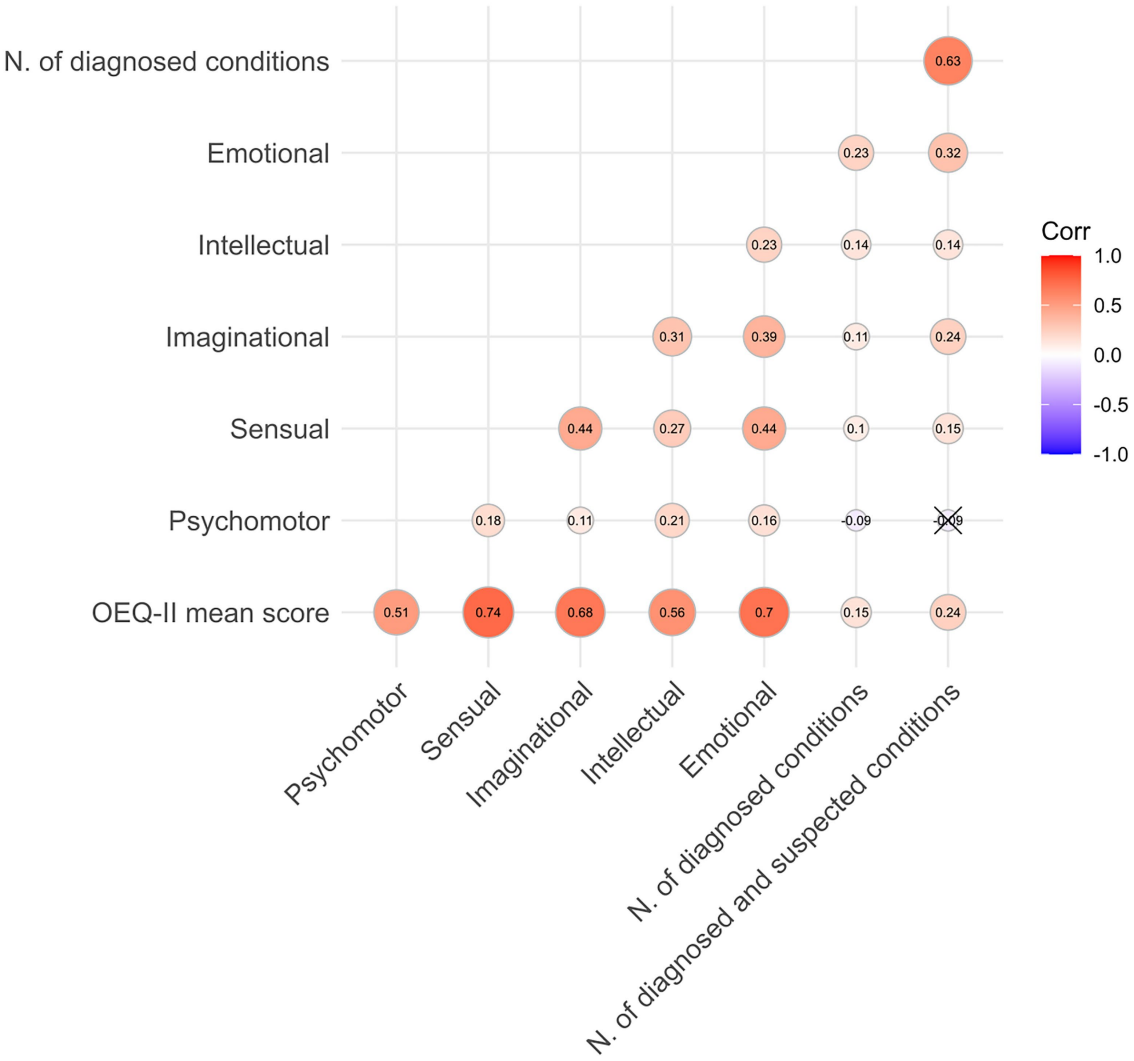
Correlation heatmap for bivariate Spearman correlations of the number of physiological and psychological overexcitabilities and factor scores from the OEQ-II.

The overexcitability concept adopted in the current study as well as by Karpinski et al. (2018) departed from Dąbrowski’s notion of overexcitability. One of our objectives was to investigate how these two interpretations of overexcitability are linked. To this end, we examined associations of the OEQ-II, which is based on Dąbrowski’s theory, with the number of reported physical and mental health conditions (see Figure 4 for a correlation heatmap of bivariate associations of OEQ-II scores and the number of reported conditions). OEQ-II mean score exhibited a small correlation with the number of diagnosed conditions (*r* = 0.15, *p* < 0.01) and a slightly stronger one with combined conditions (*r* = 0.24, *p* < 0.01).

Among OEQ-II factor scores, emotional overexcitabilities showed the strongest correlations with the number of diagnosed conditions (*r* = 0.23, *p* < 0.01) as well as with the number of combined conditions (*r* = 0.32, *p* < 0.01).

## 4. Discussion

In this sample of intellectually gifted members of MENSA, we found elevated rates of diagnosed Asperger’s syndrome, autism, cancer, chronic fatigue syndrome, depression, generalized anxiety, and irritable bowel syndrome compared to the general population. This indicates a pattern of mental health challenges that appears to be specifically prevalent in this particular group of intellectually gifted individuals.

In terms of effect sizes, Asperger’s syndrome exhibited the largest effect among diagnosed conditions in this study (*RR* = 98.55). Not considered a clinical disorder by itself, Asperger’s syndrome is a condition that shares some aspects with autism but does not entail the more dysfunctional features such as a delay in cognitive development (Tarazi et al., 2015).

General autism also exhibited considerably elevated rates. In total, more than 5 percent of the MENSA sample reported an Asperger’s or autism diagnosis. ASDs have been described as conditions of high intelligence coupled with low emotional and social competence (Alvares et al., 2020). Here, we also observed an elevated prevalence of autism in a sample of gifted individuals. However, the average IQ among autism spectrum disorders is below the general population’s IQ. Researchers have explained this discrepancy by demonstrating that autism often comes with imbalanced components of intelligence, some being highly developed while others are functioning poorly (Crespi, 2016). Here, we sampled exclusively in the upmost percentiles of the intelligence distribution and only captured the high-IQ proportion of the population of individuals with autism spectrum disorders. The prevalence of ASDs in the intellectually gifted is still a subject of scholarly debate. Recent evidence indicates a positive genetic correlation between ASD and cognitive ability (Clark et al., 2016) while other studies find a negative association (e.g., Van Eeghen et al., 2013). Our results tend to favor the former conclusion, but more work is needed to shed light on the explanatory mechanisms behind the association. Of note, the diagnostic category “Asperger’s syndrome” has been criticized for its lack of distinctness from general autism (Ghaziuddin, 2010). It has been replaced by the term “autism spectrum disorders” in the latest iterations of both the Diagnostic and Statistical Manual of Mental Disorders (DSM-V; e.g., Volkmar et al., 2021) and the International Classification of Diseases and Related Health Problems (ICD-11; e.g., Reed et al., 2019). Here, we chose to include Asperger’s syndrome nonetheless because it had been referenced in the original study which we aimed to replicate (Karpinski et al., 2018).

In the current sample, cancer rates were somewhat elevated. High intelligence is well established as a predictor of favorable health, which is also reflected by low rates of risk-behavior related types of cancer (Deary et al., 2021). In this study, 19 persons reported a cancer diagnosis (3.09 percent) which led to a *RR* of 3.07. We did not differentiate between specific forms of cancer. Karpinski et al. (2018) proposed that intellectually gifted individuals are at a higher risk of physical and mental illness due to a heightened state of awareness and alertness resulting in chronic stress. Subsequently, a series of immunological events is triggered that causes various physical and mental health conditions (Karpinski et al., 2018). Recent evidence indicates that autoimmunity is a relevant factor in the genesis of tumors (Li and Chen, 2021). However, the present sample prevalence of autoimmune diseases was only slightly and not statistically significantly elevated which contradicts a psychoneuroimmunological explanation for the elevated cancer prevalence.

One single case of ALS was reported in our sample. This was sufficient to yield a risk ratio greater than 18 because the population prevalence is remarkably low (0.01 percent).

Chronic fatigue syndrome is a condition that causes chronic exhaustion without a detectable underlying physical explanation (Bansal et al., 2012). Exposure to constant stress has been proposed as a contributing factor in its etiology (Holgate et al., 2011). Levels of generalized anxiety and depression were also significantly elevated in this sample, which might indicate that intellectually gifted individuals are indeed more susceptible to stressors and therefore more vulnerable for stress-related conditions, such as chronic fatigue syndrome. Further support for a stress-related mechanism is provided by an elevated sample prevalence of irritable bowel syndrome which is closely coupled to stressful life events (Chey et al., 2015).

Following Dąbrowski’s theory of positive disintegration, intellectual giftedness comes with a higher susceptibility to external stimuli which can subsequently elicit increased rumination and worry (Dąbrowski, 1966). Depression can be the psychopathological outcome of rumination (Marchetti et al., 2012), and we observed a substantially elevated prevalence in the current sample, which is in line with both Karpinski et al.’s (2018) as well as Dąbrowski’s concept of overexcitability.

Contrary to Karpinski et al.’s (2018) findings, we encountered neither elevated rates of diagnosed allergies, asthma, nor autoimmune diseases. The authors suggested that allergies, autoimmune diseases, and ASDs are the results of a chain of immunological events that are triggered by rumination and worry to which intellectually gifted individuals are particularly prone. However, our results do not replicate these findings, because only a limited number of the expected prevalences were elevated in the present sample. The proposed psychoneuroimmunological mechanism cannot be held solely responsible for these conditions.

### 4.1. Diagnosed vs. suspected conditions

Following the method of Karpinski et al. (2018), we included a combined conditions category, because they suggested that many conditions may go unnoticed due to individuals never getting properly diagnosed. When we combined diagnosed and suspected conditions, rates increased considerably across all conditions, with Asperger’s syndrome showing an exceptionally elevated risk (*RR* = 399.11). An analogous pattern was observed by Karpinski et al. (2018), albeit less pronounced in the case of Asperger’s.

However, the combined category must be interpreted with caution. None of the studies that Karpinski et al. (2018) used as reference points for their comparisons—and none of the studies that we used—included suspected conditions in their prevalence estimates. Moreover, the absence of population reference values for the combined category impedes its interpretation. Nonetheless, we decided to include suspected conditions to allow meaningful comparisons with reported values of Karpinski et al. (2018).

Although self-reports of suspected conditions may be considered proxies for the true prevalence, which in turn may be underestimated due to undiagnosed cases, we suggest that health anxiety (HA) could influence response behavior in suspected conditions. HA, sometimes referred to as hypochondria, predisposes individuals to interpret physical sensations or minor bodily changes as signs of a disease (Asmundson et al., 2010). Thus, a person afflicted by HA is likely to report higher levels of suspected conditions, and therefore the combined category may be reflective of this. Research suggests that the prevalence of HA in clinical practice may be as high as 20 percent of patients (Tyrer et al., 2011).

HA has also been found to substantially correlate with generalized anxiety and depression (e.g., Singh and Brown, 2014). Here, we found that the HEXACO personality factor Emotionality—which includes anxiousness—correlated more substantially with combined conditions, compared to its correlation with diagnosed conditions only. This was especially evident on facet level. Anxiety exhibited a substantially larger correlation with combined conditions compared to diagnosed conditions only (*r* = 0.37 vs. *r* = 0.23). This could also explain why correlations with certain strategies for coping with emotionally stressful situations are larger for combined conditions compared to diagnosed conditions. Individuals suffering from HA are more likely to report more suspected health conditions. These individuals could also be more likely to be afflicted by anxiety and depression which, in turn, correlate more substantially with avoidant and emotion-focused coping (e.g., self-blame or self-distraction), but less substantially with problem-focused coping (Seiffge-Krenke and Klessinger, 2000). However, a causal direction of this effect cannot be established in the current correlational design.

There is little research on the prevalence of HA in intellectually gifted individuals. Given the ruminative tendencies exhibited by many gifted persons (Du Pont et al., 2020), elevated rates of HA seem plausible. However, empirical studies are needed to support this hypothesis.

### 4.2. Is overexcitability a useful concept?

In this study, our secondary goal was to identify the degree to which the overexcitability concept of Karpinski et al. (2018) overlaps with the original meaning of the term introduced by Dąbrowski (1966). On a larger scale, we intended to establish whether this novel categorization of physical and mental health conditions is a useful addition to the scientific field. We correlated the number of reported health conditions with OEQ-II mean and factor scores. The number of diagnosed conditions was only moderately associated with the OEQ-II mean score. On the factor scores level, diagnosed conditions exhibited modest correlations with emotional overexcitabilities. Similar to coping behavior, correlations were higher when the number of combined conditions was used in analyses. We suggest that HA also contributed to the higher correlations for the combined conditions category.

Our results suggest that Karpinski et al.’s overexcitabilities concept is only moderately associated with Dąbrowski’s theory of positive disintegration. The HEXACO, a larger-scale model of personality, appeared to be better suited to explain the associations. Based on these findings, we suggest that “overexcitabilities” might not be a suitable term for the phenomenon discussed here. In all, the modified overexcitabilities concept is considerably removed from the original construct (Dąbrowski, 1966) which has also been criticized in terms of construct proliferation. Investigations of overexcitability as interpreted within the Theory of Positive Disintegration have concluded that it has hardly any incremental explanatory value beyond personality in characterizing gifted individuals (e.g., Vuyk et al., 2016a,b).

### 4.3. Limitations

First and foremost, the study sample was recruited from members of various chapters of the MENSA society, the world’s largest association of intellectually gifted individuals. While sampling in this group has its advantages, such as a professionally performed assessment of participants’ cognitive abilities, participant self-selection is an obstacle towards generalizability. Membership in MENSA is unlikely to be equally attractive to all intellectually gifted individuals. Moreover, many gifted persons never seek admission into a high-intelligence society. Therefore, MENSA itself is unlikely to be representative of the entirety of the gifted population, thus strongly limiting the generalizability of the results. However, there is very little evidence on the characteristics of MENSA members, and apart from the study we attempted to replicate (Karpinski et al., 2018), none of the existing studies are recent (e.g., Taft, 1971). Thus, more work is needed to understand in which respects members of MENSA differ from the rest of society.

Second, a general problem of online surveys, amongst others, pertains to potential socially desirable responding, lacking attention, or uncertainty whether the intended recipient themselves or rather somebody else responded to the survey.

Generally, it proved challenging to find appropriate population prevalence estimates for many health conditions. First, diagnostic criteria are not always consistent across studies, leading to varying results. For some conditions, multiple definitions exist, which are at times in contradiction with one another. Thus, prevalence estimates have an inherent margin of error due to varying diagnostic procedures, definitions, and study methodologies. Here, we chose to select the largest available study for each condition that matched the construct definition as well as the geographical region of our participants. Using this approach, we were able to carefully survey studies and exclude inappropriate prevalence estimates. Nevertheless, in some cases uncertainty prevails. Asperger’s is notoriously elusive and difficult to distinguish from high-functioning autism (Planche and Lemonnier, 2012). This makes it challenging to establish a robust estimation of the population prevalence, and few studies have attempted this. Thus, the population prevalence of 0.03 percent to which our sample prevalences were compared may be an imprecise estimation of the true population prevalence.

### 4.4. Future directions

As the current study is a replication attempt, we reproduced the methodology of the original study as closely as possible. However, an alternative approach to handle the problem of inconsistent prevalences is to carry out a case–control study. Each gifted participant is assigned a control from the general population that is matched in geographical region, age, sex, and sociodemographic characteristics. Both gifted and control are asked to take the same questionnaire. We recommend that future research be carried out using such an approach as a complimentary method.

Here, we adopted a purely intelligence-based definition of giftedness. We suggest that future studies consider broader giftedness concepts to investigate how potential health effects map onto different components of giftedness, such as achievement motivation (Tannenbaum, 2003), creativity (Renzulli, 2005), or well-being (e.g., Drigas et al., 2017).

### 4.5. Conclusion

Here, we demonstrate that intellectually gifted individuals, sampled from a high-IQ society that requires an IQ score above 130 for membership, face specific health challenges. However, our results indicate that the nature of these challenges seems to be rooted in mental rather than physical health, although potential effects of sample selectiveness must be considered and need further investigation. In addition, we suggest that these challenges should not be summarized using the term “overexcitabilities” because they are only minimally related on a conceptual and empirical basis.

## Data availability statement

The raw data supporting the conclusions of this article will be made available by the authors, without undue reservation.

## Ethics statement

Ethical review and approval was not required for the study on human participants in accordance with the local legislation and institutional requirements. The patients/participants provided their written informed consent to participate in this study.

## Author contributions

JF, TB, KK, and JP: conceptualization and methodology. JF: software, formal analysis, investigation, data curation, writing—original draft preparation and visualization. TB, KK, and JP: writing—review and editing. JP: supervision. All authors contributed to the article and approved the submitted version.

## Funding

KK received funding by the National Research, Development and Innovation Office of Hungary: Grant FK-21-138971, by the János Bolyai Research Scholarship of the Hungarian Academy of Sciences and by the ÚNKP-22-5 New National Excellence Program of the Ministry for Innovation and Technology from the source of the National Research, Development and Innovation Fund.

## Conflict of interest

The authors declare that the research was conducted in the absence of any commercial or financial relationships that could be construed as a potential conflict of interest.

## Publisher’s note

All claims expressed in this article are solely those of the authors and do not necessarily represent those of their affiliated organizations, or those of the publisher, the editors and the reviewers. Any product that may be evaluated in this article, or claim that may be made by its manufacturer, is not guaranteed or endorsed by the publisher.

## Footnotes

^1^ https://doi.org/10.17605/OSF.IO/5U6NM
